# COVID-19 risk perception and hoax beliefs in the US immediately before and after the announcement of President Trump's diagnosis

**DOI:** 10.1098/rsos.212013

**Published:** 2022-08-03

**Authors:** Lisa-Maria Tanase, John Kerr, Alexandra L. J. Freeman, Claudia R. Schneider

**Affiliations:** ^1^ Winton Centre for Risk and Evidence Communication, University of Cambridge, Cambridge, UK; ^2^ Department of Psychology, University of Cambridge, Cambridge, UK

**Keywords:** COVID-19, misinformation, risk perception

## Abstract

A notable challenge of the SARS-CoV-2 pandemic has been public scepticism over the severity of the disease, or even its existence. Such scepticism is politically skewed in the USA, with conservatives more likely to downplay or deny the risks of the virus. However, the hospitalization of President Trump with COVID-19 in October 2020 served as a high-profile exemplar of the reality and risks of the virus, and as such may have influenced opinions, particularly for US conservatives. We investigate whether President Trump testing positive was associated with changes in public attitudes towards the virus. In two studies, we surveyed independent representative US samples before and after the announcement of Trump's illness. In Study 1, measuring risk perceptions of the virus, we find that participants surveyed before and after the announcement did not differ in their risk perception regardless of political orientation. In Study 2, measuring belief that the virus is a hoax, we find that among those on the far right of the political spectrum, hoax belief was lower for those surveyed after the announcement, suggesting that Trump's hospitalization may have affected the beliefs of those most receptive to the President's earlier suggestions that the virus might be a hoax.

## 1. Introduction

In the words of the Director of the World Health Organization, ‘We're not just fighting an epidemic; we're fighting an infodemic’ [1]. At times when information is uncertain or rapidly changing, conflicting information can arise, which can affect people's risk perception and undermine their trust and belief in the necessity for, or efficacy of, behavioural recommendations and guidelines. This can worsen an epidemic by reducing protective behaviours taken by individuals. Scepticism about both the existence and severity of SARS-CoV-2 have been associated with lower compliance with COVID-19 protective and preventive behaviours, including physical distancing, use of face masks, and vaccination intention [2,3], and lower risk perception [4]. Indeed, the very features defining a crisis such as the SARS-CoV-2 pandemic have been suggested as increasing susceptibility to misinformation and distorted risk perceptions. These features are: uncertainty and novelty, rapid onset, potentially severe losses, apparent randomness, and a lack of control [5,6]. These elements are then coupled with information overload. Kaufhold *et al*. [7] emphasized that large amounts of information during large-scale crises can lead to information levels exceeding cognitive processing capacity, at which point decision quality diminishes. Under these circumstances, more intuitive natural assessments and heuristics act as rules of thumb [8].

One form of cue particularly likely to act as a powerful heuristic influencing risk perception and COVID-19-related beliefs is the use of ‘exemplars' [9], i.e. individual case reports that illustrate and simplify complex ideas. Research has previously shown the potential effects of such exemplars on health attitudes [10,11], including increased issue awareness, health-related communication, engagement in prevention and treatment, and reduced disease stigmatization [12–15]. There is also evidence that exposure to exemplars about threats can magnify perceptions of personal susceptibility to, and severity of, the hazard in a variety of domains (e.g. [9,16,17]). As such, it might be expected that news reporting of well-known public figures may influence people's perception of the risk of COVID-19 because they tap into both availability [18] and representativeness heuristics [8]. The availability heuristic—i.e. people tending to evaluate the probability of events by the ease with which relevant instances come to mind—could lead certain individuals to see the risk of COVID-19 as more salient following high coverage of well-known public exemplars. Similarly, the representativeness heuristic—i.e. people estimating the likelihood of an event by comparing it with an existing prototype that already exists in their mind—may enhance risk perceptions around the virus following a vivid case of a public figure.

Some evidence already exists to support this hypothesis. A mixed-methods study [19] found that participants who had been exposed to the news of celebrity Tom Hanks being diagnosed with COVID-19 reported attitude changes, stressing it ‘highlighted the reality of COVID-19' (p. 828), increasing their perception of both the severity of the situation and personal risk from the illness.

In this work, we build on this evidence to investigate the question of whether the experience of a well-known figure who featured in the media as suffering from illness as a result of infection is associated with changes in public attitudes to, and perceptions of, the virus.

**Figure 1.**
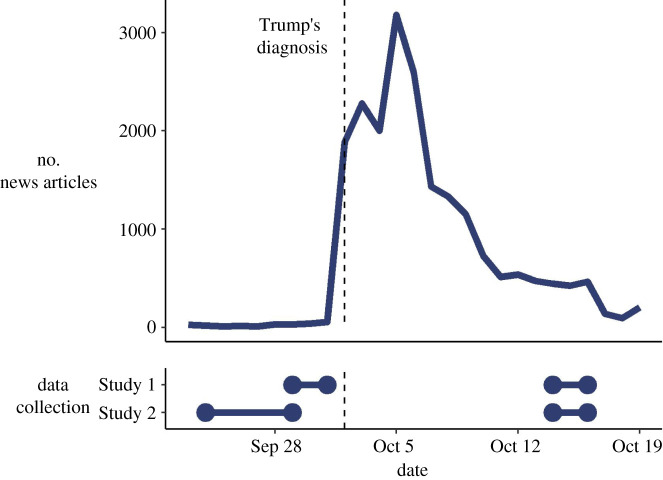
Timeline of media coverage and data collection before and after President Trump tested positive for COVID-19.

On 2 October 2020, it was announced that the incumbent President of the USA, Donald Trump, a potentially influential denier of COVID-19 [20,21], had tested positive for SARS-CoV-2 and had been subsequently hospitalized with COVID-19 symptoms [22]. This provided an almost natural experiment with which to study the relationship between a salient exemplar and attitudes about COVID-19. Fortuitously, in two separate studies, we collected survey data from US participants measuring risk perception (in Study 1) and hoax beliefs (in Study 2), in the days before and after President Trump was hospitalized with the virus. This allowed us to undertake an exploratory analysis comparing these variables in the two waves of each survey. Figure 1 provides a timeline of relative media interest in the event, and the periods of data collection before and after the announcement for the two studies. News article data was drawn from the media database Factiva (Dow Jones, New York, NY, USA), based on daily counts of articles from US news sources mentioning ‘COVID' or ‘Coronavirus', and ‘hospital*' or ‘diagnos*' in the same paragraph as ‘Trump'.

We also set out to investigate the role of political affiliation within our study samples. Following from prior research, we expected that, overall, individuals who identify as more conservative or as Republicans would perceive the virus to be of lower risk and be more likely to endorse the claim that the virus is a hoax. During the pandemic, Trump predominantly downplayed the risk of the virus [21] and he repeatedly likened the threat to the common ‘flu’ [23]. In the early days of the pandemic, President Trump referred to COVID-19 as ‘their [Democrats'] new hoax', although there was dispute about what precisely he had meant by the phrase [24]. A survey by the Pew Research Center found that US residents who relied upon President Trump and his task force for information around COVID-19 were more likely to believe that the risk from the disease was overestimated [25]. Similarly, in counties with more Trump voters, residents were less prone to look for coronavirus information or respect physical distancing [26], and a study examining the association between political ideology and perceptions of COVID-19 found that right-wing political orientation was associated with lower perceived virus severity and greater beliefs that the spread of the virus was a conspiracy [27].

While it seems that conservatives are, on average, more sceptical about the virus, they may also be more likely than their liberal counterparts to shift their beliefs in response to the exemplar of Trump's much publicized diagnosis. The clearest evidence for this comes from the previously cited study examining the impact of Tom Hanks' COVID-19 diagnosis. The authors report that those participants who identified with Tom Hanks, in particular, were more likely to report changes in their thoughts or COVID-related behaviours following the announcement [19]. In a similar vein, we might expect US residents who identify more with President Trump (e.g. conservatives and Republicans) to be more likely to be influenced by the announcement of his diagnosis and to increase their perception of the risk and/or decrease their perception of COVID-19 being a hoax. In the words of a Trump supporter in Ohio: ‘To see he has it wakes you up a bit. Anyone can get it, even the president.' [28].

In two exploratory studies, we compared COVID-19 risk perception and hoax beliefs in the US public before and after President Trump's diagnosis, and investigated the potential role of political affiliation as a moderator.

It was not possible to formulate hypotheses prior to data collection, but we frame our exploratory research as addressing the following research questions. Did US residents' COVID-19 risk perceptions (Study 1) or hoax beliefs (Study 2) change following the announcement that President Trump had been diagnosed with COVID-19? And did potential changes vary depending on political orientation?

We expected that we would find increased COVID-19 risk perception and decreased beliefs of COVID-19 being a hoax among the public surveyed after the President's diagnosis, and that this difference might be greater among participants identifying themselves as politically Republican or conservative. As there is evidence that factors such as education [29,30], gender [31,32], and age [32,33] play a consistent and important role in influencing risk perception and conspiracy beliefs, particularly in relation to health, we controlled for key demographics in our models.

### 1.1. Study context

Both studies drew on independent representative US samples surveyed before (wave 1) and shortly after (wave 2) the announcement of Trump's diagnosis.

Notably, all waves were quota-sampled to be representative of the US population in terms of age and gender (and ethnicity in Study 1). In this manner, we serendipitously collected data on COVID-19 beliefs among US residents just before and after the announcement that President Trump had been diagnosed with COVID-19. Both studies were part of a larger set of studies carried out by the research team as part of the Systematizing Confidence in Open Research and Evidence (SCORE) project. Sample sizes were thus set by the larger project, not the research presented here. Furthermore, several of the survey measures used in the research presented here were designed as part of the larger project. We remind the reader that due to the unexpected nature of Trump's diagnosis during data collection, it was not possible to pre-register hypotheses or analyses before data collection. Therefore, the current studies should be considered exploratory rather than confirmatory. For more information on that project, including all measures collected in the surveys, please visit the public project repository here: https://osf.io/agztm/.

## 2. Study 1: risk perception and President Trump's diagnosis

### 2.1. Methods

#### 2.1.1. Participants and procedure

Participants residing in the US were recruited through the panel provider Prolific (prolific.co), which provided national quota samples stratified by age, gender, and ethnicity. Wave 1 was run between 28 September and 1 October 2020 and included 909 participants; wave 2, run between 14 and 16 October 2020 included 447 participants (participants from wave 1 were excluded from wave 2), giving a total sample of *N* = 1356. These sample sizes were determined by the SCORE replication project, as noted above. A *post-hoc* sensitivity analysis conducted using G*Power [34] indicated that the sample size was sufficient to detect a change in slopes between groups of *β* = 0.11, at 0.80 power and *α* set at 0.05. Participants provided informed consent and completed the survey on the Qualtrics survey platform.

#### 2.1.2. Measures

All measures were consistent across both waves. Of relevance to the current study, participants completed a six-item COVID-19 risk perception index encompassing cognitive, affective, and temporal-spatial dimensions of risk perception, (example item: ‘Getting sick with the coronavirus/COVID-19 can be serious'; *α* = 0.81) [4,31]. This measure of risk perception has been used in prior research where it showed good inter-item reliability [4,31]. It is designed to capture risk perception as a more ‘holistic' concept rather than focusing solely on its affective or cognitive components. See electronic supplementary material for a full list of the items of the risk perception index. We also collected socio-demographic variables, including gender (binary: male, female), age, self-reported political orientation (1 = very left wing/liberal, 7 = very right wing/conservative), and education level (self-reported, ranging from 1 = ‘no formal education above 16' to 7 = ‘PhD').

The survey additionally included several other COVID-19-related items that were part of the larger replication project but not the focus of the current study.

#### 2.1.3. Analysis

We used hierarchical linear regression modelling to investigate a potential association between President Trump's diagnosis and COVID-19 risk perception, as well as the role of political orientation and socio-demographic factors. We first regressed risk perceptions onto wave, political orientation, and demographic factors, testing whether risk perceptions differed between participants recruited before and after the announcement of Trump's diagnosis, controlling for political orientation and demographic factors. We then tested for a potential interaction between wave and political orientation, i.e. whether any observed differences in risk perception between waves were dependent on political orientation. All analyses were conducted in R v. 4.0.3 (10 October 2020).

### 2.2. Results

Table 1 reports the results of the regression models (descriptive statistics and bivariate correlations are shown in the electronic supplementary material). Controlling for political orientation and socio-demographic factors, we found no significant association between wave and risk perception (Model 1), indicating that there was no significant difference in risk perception between participants recruited before and after announcement of Trump's COVID-19 diagnosis in our sample. Adding the interaction term in Model 2 did not change the picture; there was no significant interaction between wave and political orientation.

**Table 1.** Hierarchical regression results for COVID-19 risk perception.

	COVID-19 risk perception	
	(1)	(2)
intercept	4.77***	4.73***
	(4.43, 5.11)	(4.37, 5.08)
gender(female)	0.24***	0.23***
	(0.12, 0.35)	(0.12, 0.35)
age	0.005*	0.005*
	(0.001, 0.01)	(0.001, 0.01)
education	0.12***	0.12***
	(0.07, 0.17)	(0.07, 0.17)
political orientation	−0.21***	−0.20***
	(−0.24, −0.17)	(−0.24, −0.15)
wave	0.05	0.18
	(−0.08, 0.17)	(−0.15, 0.51)
wave × political orientation		−0.03
		(−0.11, 0.04)
observations	1340	1340
adjusted *R*^2^	0.12	0.12

*p < 0.05, **p < 0.01, ***p < 0.001.

Unstandardized estimates (95%CI) shown.

However, political orientation emerged as a significant predictor in Model 1, showing that the further right participants were on the political spectrum, the lower their risk perception. Older participants, more highly educated participants, and females expressed, on average, higher risk perception.

### 2.3. Discussion

We did not find evidence that news of President Trump's COVID-19 diagnosis was associated with differences in US residents' risk perception, regardless of their position on the political spectrum. A possible explanation for this lack of effect may be the way in which the president framed his announcement. Despite admitting his COVID-19 infection, President Trump publicly projected the image of someone not anxious about the disease and optimistic about his recovery [35]. This framing may have projected little difference from his previous statements about the virus, which had already influenced many of his followers in believing that the risk from the virus was being overestimated [25]. The fact that the 74-year-old president fell into a high-risk category for COVID-19 may also have led many US residents (in lower risk groups) to feel no need to change their opinion about the risk posed by the disease following President Trump's diagnosis.

The finding that political orientation overall was a significant predictor of risk perception indicates that political factors are associated with COVID-19 risk perception in the USA. In this instance, the more right-leaning participants tended to have lower risk perception, controlling for effects of age, gender, education, and wave of data collection. This finding is in line with previous research showing that being on the right wing of the political spectrum is associated with lower perceived virus severity [27] and lower risk perception [31]. Our findings regarding the role of socio-demographic factors in risk perception also link to previous research, i.e. higher risk perception for more educated participants [29,30], females [4,31], and older participants [32,33].

## 3. Study 2: hoax belief and President Trump's diagnosis

Study 2 presented an opportunity to explore whether there was a difference between US residents' beliefs about the pandemic being a hoax before and after President Trump's diagnosis, controlling for demographic factors. In Study 2, we also controlled for general susceptibility to misinformation, in order to focus on COVID-19 hoax beliefs independently from a more general propensity to believe misinformation. However, analysis results did not change when susceptibility to misinformation was not included in the model (see electronic supplementary material).

### 3.1. Methods

#### 3.1.1. Participants and procedure

US residents were recruited for the study through the ISO-certified online panel provider Respondi and quota sampled to be representative of the US population in terms of age and gender. Participants who reported not paying attention (*n* = 96) in response to an attention check item did not count towards quotas and were excluded from analyses (repeating analyses with these participants included did not substantially alter results, see below). The resulting sample size was 949 participants for wave 1 (collected between 24 and 29 September 2020) and 1191 participants for wave 2 (collected between 14 and 16 October 2020), amounting to a total sample of *N* = 2140. As for Study 1, sample sizes for both waves were determined by the larger project under which data were collected. A *post-hoc* sensitivity analysis conducted using G*Power [34] indicated that the sample size was sufficient to detect a change in slopes between groups of *β* = 0.09, at 0.80 power and α set at 0.05. All participants provided informed consent and completed the survey on the Qualtrics survey platform.

#### 3.1.2. Measures

All measures were consistent across both waves. Participants were asked the extent to which they believed the coronavirus pandemic to be a hoax (1 = *definitely not*, 6 = *definitely*), a measure used by Stanley *et al.* [36,37]. Socio-demographic variables collected included gender (binary: male, female), age, self-reported political orientation (1 = very left wing/liberal, 7 = very right wing/ conservative), and education level (self-reported, ranging from 1 = *Did not complete high school* to 5 = *Graduate or Professional degree*). General susceptibility to misinformation was tested using the MIST measure [38]. In this, participants were exposed to 20 news headlines (10 real and 10 fake) and asked to rate each of them as either true or false. The number of correct responses was indexed as a 0–10 measure of misinformation susceptibility.

#### 3.1.3. Analysis

As in Study 1, hierarchical linear regression modelling was used to examine the research questions. First, hoax beliefs were regressed onto wave (before or after announcement of President Trump's diagnosis), controlling for political orientation and demographic factors. Susceptibility to misinformation was then added in a subsequent model. Lastly, as in Study 1, a test for an interaction between wave and political orientation was conducted to explore whether any observed differences in hoax beliefs between waves depended on political orientation. All analysis was conducted in R v. 4.0.3 (10 October 2020).

### 3.2. Results

**Figure 2.**
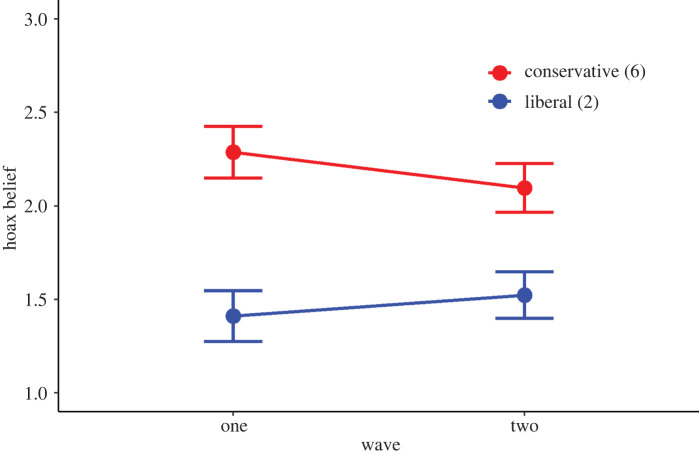
Interaction effects between wave and political orientation. Estimated marginal means and 95%CI shown. For visualization purposes, the figure shows levels of hoax belief across waves for liberals and conservatives using answer points 2 and 6, respectively, on the political orientation answer scale used in the surveys. A visualization showing the values of political orientation at which the effect of wave on hoax belief is statistically significant (Johnson–Neyman interval) is provided in the electronic supplementary material.

Results from the regression models are reported in table 2. Considering only wave and demographic factors and political orientation as control variables (Model 1), as well as when including susceptibility to misinformation as an additional predictor (Model 2), we did not find a significant effect of wave of recruitment (before or after Trump's COVID-19 announcement) on hoax beliefs. However, we observed a significant interaction between wave and political orientation in Model 3. This interaction was also significant in a model that did not control for susceptibility to misinformation (see electronic supplementary material, table S7). The interaction indicates that among participants reporting left-wing or liberal political views, there was no difference between waves. However, among more conservative/right-wing participants, we find that endorsement of hoax claims was lower for those surveyed after Trump's announcement, compared with before (figure 2). To examine at which point(s) on the political spectrum a significant difference between waves emerged, a Johnson–Neyman analysis was conducted [39]. We report a Johnson–Neyman interval of [−1.27, 5.25], such that the effect of wave in the interaction model becomes significant when political orientation is outside this interval. This indicates that wave has a significant (*p* < 0.05) effect on hoax belief among those reporting conservative (6) or very conservative (7) political views on our answer scale. There was no difference between waves for liberals (see electronic supplementary material, figure S1 for an illustration of the Johnson–Neyman interval).

**Table 2.** Hierarchical regression results for COVID-19 hoax belief.

	belief in COVID-19 being a hoax		
	(1)	(2)	(3)
constant	1.65***	0.77***	0.61***
	(1.32, 1.97)	(0.46, 1.09)	(0.26, 0.96)
gender (female)	−0.11*	−0.16**	−0.16**
	(−0.23, −0.0003)	(−0.27, −0.05)	(−0.27, −0.05)
age	−0.02***	−0.01***	−0.01***
	(−0.02, −0.01)	(−0.01, −0.01)	(−0.01, −0.01)
education	0.005	0.06**	0.06**
	(−0.04, 0.05)	(0.02, 0.11)	(0.02, 0.11)
political orientation	0.28***	0.18***	0.22***
	(0.24, 0.31)	(0.14, 0.21)	(0.17, 0.27)
wave	−0.03	−0.04	0.26
	(−0.15, 0.08)	(−0.15, 0.07)	(−0.02, 0.54)
misinformation susceptibility		0.30***	0.30***
		(0.27, 0.33)	(0.27, 0.33)
political orientation × wave			−0.08*
			(−0.14, −0.01)
observations	2123	2123	2123
adjusted *R*^2^	0.11	0.23	0.23

*p < 0.05, **p < 0.01, ***p < 0.001.

Unstandardized estimates (95%CI) shown.

Additionally, the results indicate a significant association of political orientation and susceptibility to misinformation overall with hoax beliefs, such that higher conservative/right-wing leanings and higher susceptibility to misinformation generally are associated with higher hoax beliefs. We also find a significant effect of age and gender, such that hoax beliefs are higher for younger and male participants.

Belief that COVID-19 was a hoax was skewed in the overall sample, with most participants having low belief in COVID-19 being a hoax. Therefore, we supplemented our analysis by fitting an interaction model in which log-transformed hoax belief was regressed onto predictors. This resulted in the same pattern of significant effects, supporting the robustness of the findings. We additionally repeated the analysis including attention-check failures as another robustness check. The same pattern of results was found (robustness-check analyses are available in electronic supplementary material, table S7).

As a further robustness check, we repeated analyses using reported party affiliation (Democrat, *n* = 775; Republican, *n* = 701; independent, *n* = 668) rather than liberal/conservative orientation as a measure of political views. Results were consistent: Republicans in wave 2 expressed lower belief that COVID-19 is a hoax than Republicans in wave 1 (controlling for other factors). There was no significant difference across waves for Democrats or independents (see electronic supplementary material, figure S2 and tables S7 and S8).

### 3.3. Discussion

Our data did not show evidence of hoax beliefs being significantly different between waves overall; however, we did find a significant interaction. Regression results suggested that leaning further to the right on the political spectrum was associated with lower belief in COVID-19 being a hoax after the announcement that President Trump had tested positive, compared with beforehand, controlling for participants' domain-general susceptibility to misinformation, age, gender, and education. Left-leaning participants, meanwhile, exhibited little difference in their belief about COVID-19 being a hoax between waves. In the same way that those more likely to change their attitudes following Tom Hanks' COVID-19 diagnosis were participants who most identified with him [19], it seems likely that politically right-leaning participants in the current study identified more closely with President Trump, making his exemplar more vivid and influential in their mind and hence having more influence on their beliefs, in line with previous literature on exemplars [19].

The finding that general susceptibility to misinformation was a significant predictor of hoax beliefs suggests that general susceptibility to misinformation may spill over into the COVID-19 domain. This is in line with previous findings that predisposition to any form of misinformation is an important factor driving politically motivated reasoning [40]. Our finding that more right-wing political orientation overall was associated with higher hoax beliefs is in line with prior research that has shown political conservatism is associated with belief that the spread of the virus was a conspiracy [27]. Lastly, the finding that demographic variables, such as being younger and male, are associated with higher hoax beliefs likewise is in line with the prior literature [32,33].

## 4. General discussion

Across two surveys conducted between September 2020 and October 2020, we were able to investigate US residents' attitudes to COVID-19 and, fortuitously, potential differences between participants surveyed before and after the announcement that President Trump had been diagnosed with the disease.

Comparing participants surveyed before and after President Trump's COVID-19 diagnosis, we did not find a statistically significant difference in risk perception, but we did find a statistically significant difference in beliefs in the virus being a hoax in politically right-leaning participants. Our data suggest that belief that COVID-19 is a hoax decreased among this group following the announcement of President Trump's diagnosis. This finding is in line with previous research pointing at the importance of exemplars for people with congruent views in influencing their attitudes and perceptions [19]. That is, the highly publicized example of a high-profile individual experience can shift the beliefs of the public, particularly of those who identify more with the individual.

Our findings suggest that President Trump's diagnosis may have played a role in shifting some US residents' beliefs in COVID-19 being a hoax, but not their risk perception related to the virus. This highlights a difference between believing that the virus is not serious and believing that the virus isn't real at all. Although President Trump tested positive, he claimed in the media that he did not feel threatened by his infection (despite being at higher risk of adverse outcomes due to age) [41]. Thus, some supporters of Trump may have become more accepting of the reality of the virus (i.e. that it is not a hoax), while maintaining a low level of perceived risk. Support for the possibility that President Trump's influence may have had differential effects on beliefs that COVID-19 is a complete hoax, and beliefs that it is a disease but not serious, can be found in a recent Twitter analysis study [42]. This research reported the proportion of tweets claiming that (1) the virus was not real, and (2) the virus was not serious. Before President Trump tested positive, 19.5% of tweets claimed that COVID-19 was a hoax and 13.3% that it was not serious, but after his diagnosis, only 3.1% and 1.4% did, respectively, which would suggest a holistic change across both domains. However, after the president subsequently tweeted ‘Don't be afraid of COVID-19', this effect was slightly reversed, with 10.0% of tweets claiming the virus was a hoax and up to 12.0% claiming it was not serious. Thus, it is possible that the way in which the President framed his infection may have attenuated any positive impact on risk perception while still decreasing the belief in COVID-19 being a hoax. Indeed, President Trump's tweet announcing he and his wife had tested positive for COVID-19, were quarantining, and had started their recovery process became Trump's most retweeted tweet ever [43]. It is conceivable that this tweet—openly and publicly acknowledging his diagnosis after having appeared more sceptical in the past [20]—may have sent a strong ‘elite cue' to his supporters around the existence of the virus. Downs [44] first referred to elite cues to describe the phenomenon by which the average citizen will ‘seek assistance from men who are experts in those fields, have the same political goals he does, and have good judgement' as a shortcut to guide their political decisions [44, p. 233]. Trump's initial disclosure on Twitter can be contrasted with a tweet posted on the third day of his hospitalization in which he claimed that he felt very well, announced he would leave the hospital in a few days, and urged people not to be afraid of the virus [45]. This message conveyed that he did not feel his infection represented a severe risk to his health [41]. These elite cues that President Trump gave via his tweets may help explain our findings of no significant difference in risk perception but a significant change in hoax beliefs.

Our data also shows that, consistent with previous research [27,31], political orientation was associated with both risk perception and belief in COVID-19 being a hoax. While risk perception was relatively high on average, and belief in COVID-19 being a hoax was low, across the time period of data collection, participants identifying themselves as politically left-leaning had lower belief in COVID-19 being a hoax and higher perception of the risk of the virus than those who identified themselves as politically right-leaning. We also replicated prior findings regarding some known predictors of risk perception and conspiracy beliefs [4,29–33,46]. The demographic triad of gender, age, and education all had a significant effect on COVID-19 risk perception. Predictors of higher risk perception were being female, older, and more highly educated. Demographic predictors of higher belief in COVID-19 being a hoax across our models were being male and younger.

The finding that general susceptibility to misinformation significantly correlated with belief in COVID-19 being a hoax suggests that efforts to reduce overall misinformation susceptibility, such as inoculation [47], might also reduce belief that the virus is a hoax. As touched upon earlier, it should be noted though that belief in the virus being a hoax was low in our sample. This observation is in line with recent research that showed that, on average, susceptibility to misinformation among US participants in the early stage of the pandemic was relatively low [48]. However, political orientation was identified as a significant predictor, with Democratic participants being generally less susceptible to misinformation than conservatives [48].

Our studies have several limitations. First of all, as we could not predict Trump's diagnosis, our analytical approach was not pre-registered before data collection. However, we hope that the additional analyses reported here and in the supplementary materials, as well as the availability of the data in an open repository for further analysis, provide transparency and offer reassurance as to the robustness of our analyses and results. Secondly, the collection of data across two waves per study could only capture differences in COVID-19 attitudes days before and after President Trump tested positive, without the added granularity of a third wave following his several statements reassuring the public about his state after treatment in hospital (as in the analysis of Twitter comments cited above [42]). It is therefore possible that the lack of a statistically significant effect on risk perception was due to the time period of the study being too short, or due to President Trump indicating that he did not feel his life was in danger even when infected [41], which occurred before our second wave of data collection. Our study design also does not allow any observed differences between the two waves to be causally attributed to President Trump's diagnosis. While we controlled for a number of factors, it is possible that such differences could be attributable to different baseline beliefs between the two groups of participants, or other external factors and events unrelated to President Trump. Lastly, we also note that in Study 2, belief that the virus is a hoax was measured with a single item, and this may be a source of measurement error. Future research should consider the use of multi-item measures of such constructs to reduce measurement error (e.g. [49]).

## 5. Conclusion

We surveyed US residents shortly before and after the announcement that President Trump had been diagnosed and hospitalized with COVID-19. We find that, across the political spectrum, perceived risk of the virus did not differ between participants surveyed before and after the announcement (Study 1). However, we find that belief in the virus being a hoax was lower among politically conservative participants surveyed after the announcement, compared with before (Study 2). Our work also emphasizes the difference between perception of the risk of a threat and not believing that a threat exists at all. Although the nature of the studies does not allow us to make strong causal claims, these findings are consistent with a scenario in which the exemplar of Trump's illness shifted conservatives' beliefs about the virus being real, but not their beliefs about its potential danger.

## Data Availability

Data, code, and materials have been placed in a public project repository here: https://osf.io/agztm/. The data are provided in electronic supplementary material [50].

## References

[1] Ghebreyesus TA. 2020 Munich Security Conference. Munich, Germany: World Health Organization. See www.who.int/director-general/speeches/detail/munich-security-conference.

[2] Latkin CA, Dayton L, Moran M, Strickland JC, Collins K. 2021 Behavioral and psychosocial factors associated with COVID-19 skepticism in the United States. Curr. Psychol. **27**, 162-177. (10.1007/s12144-020-01211-3) PMC7786141 33424206

[3] Ruiz JB, Bell RA. 2021 Predictors of intention to vaccinate against COVID-19: results of a nationwide survey. Vaccine **39**, 1080-1086. (10.1016/j.vaccine.2021.01.010) 33461833 PMC7794597

[4] Dryhurst S, Schneider CR, Kerr J, Freeman ALJ, Recchia G, van der Bles AM, Spiegelhalter D, van der Linden S. 2020 Risk perceptions of COVID-19 around the world. J. Risk Res. **23**, 1466-4461. (10.1080/13669877.2020.1758193)

[5] Liu BF, Kim S. 2011 How organizations framed the 2009 H1N1 pandemic via social and traditional media: Implications for U.S. health communicators. Public Relat. Rev. **37**, 233-244. (10.1016/j.pubrev.2011.03.005)

[6] Sniezek J, Wilkins D, Wadlington P, Baumann M. 2002 Training for crisis decision-making: psychological issues and computer-based solutions. J. Manag. Inf. Syst. **18**, 147-168. (10.1080/07421222.2002.11045704)

[7] Kaufhold M-A, Rupp N, Reuter C, Habdank M. 2020 Mitigating information overload in social media during conflicts and crises: design and evaluation of a cross-platform alerting system. Behav. Inf. Technol. **39**, 319-342. (10.1080/0144929X.2019.1620334)

[8] Tversky A, Kahneman D. 1974 Judgment under uncertainty: heuristics and biases. Science **185**, 1124-1131. (10.1126/science.185.4157.1124) 17835457

[9] Aust CF, Zillmann D. 1996 Effects of victim exemplification in television news on viewer perception of social issues. J. Mass Commun. Q. **73**, 787-803. (10.1177/107769909607300403)

[10] Myrick JG. 2017 Public perceptions of celebrity cancer deaths: how identification and emotions shape cancer stigma and behavioral intentions. Health Commun. **32**, 1385-1395. (10.1080/10410236.2016.1224450) 27739882

[11] Zillmann D. 2006 Exemplification effects in the promotion of safety and health. J. Commun. **56**, S221-S237. (10.1111/j.1460-2466.2006.00291.x)

[12] Noar SM, Althouse BM, Ayers JW, Francis DB, Ribisl KM. 2015 Cancer information seeking in the digital age. Med. Decis. Mak. **35**, 16-21. (10.1177/0272989X14556130) 25349187

[13] Cohen EL. 2020 Stars—They're Sick Like Us! The effects of a celebrity exemplar on COVID-19-related risk cognitions, emotions, and preventative behavioral intentions. Sci. Commun. **42**, 724-741. (10.1177/1075547020960465)

[14] Rahmani G, McArdle A, Kelly JL. 2018 The hugh Jackman effect—the impact of celebrity health disclosure on skin cancer awareness. Dermatologic Surg. **44**, 1039-1040. (10.1097/DSS.0000000000001348) 28961637

[15] Noar SM, Willoughby JF, Myrick JG, Brown J. 2014 Public figure announcements about cancer and opportunities for cancer communication: a review and research Agenda. Health Commun. **29**, 445-461. (10.1080/10410236.2013.764781) 23845155

[16] Spence PR, Lachlan KA, Westerman D, Lin X, Harris CJ, Sellnow TL, Sellnow-Richmond DD. 2015 Exemplification effects: responses to perceptions of risk. J. Risk Res. **20**, 1-21. (10.1080/13669877.2015.1100658)

[17] Westerman D, Spence PR, Lachlan KA. 2009 Telepresence and the exemplification effects of disaster news. Commun. Stud. **60**, 542-557. (10.1080/10510970903260376)

[18] Tversky A, Kahneman D. 1973 Availability: a heuristic for judging frequency and probability. Cogn. Psychol. **5**, 207-232. (10.1016/0010-0285(73)90033-9)

[19] Myrick JG, Willoughby JF. 2021 A mixed methods inquiry into the role of Tom Hanks' COVID-19 social media disclosure in shaping willingness to engage in prevention behaviors. Health Commun. **37**, 824-832. (10.1080/10410236.2020.1871169) 33445967

[20] Strauss D, Laughland O. 2020 Trump calls coronavirus criticism Democrats’ ‘new hoax’ and links it to immigration. *Guard.* 29 February. See https://www.theguardian.com/us-news/2020/feb/28/trump-calls-coronavirus-outbreak-a-hoax-and-links-it-to-immigration-at-rally.

[21] Rutledge PE. 2020 Trump, COVID-19, and the war on expertise. Am. Rev. Public Adm. **50**, 505-511. (10.1177/0275074020941683)

[22] BBC News. 2020 Covid: Donald Trump and Melania test positive. October.

[23] Rieder R. 2020 Trump's Deceptive Comparison of the Coronavirus to the Flu.

[24] Rieder R. 2020 Trump and the ‘New Hoax’.

[25] Pew Research Center. 2020 Americans Who Rely Most on White House for COVID-19 News More Likely to Downplay the Pandemic.

[26] Barrios J, Hochberg Y. 2020 Risk Perception through the Lens of Politics in the Time of the COVID-19 Pandemic. (10.3386/w27008)

[27] Calvillo DP, Ross BJ, Garcia RJB, Smelter TJ, Rutchick AM. 2020 Political ideology predicts perceptions of the threat of COVID-19 (and susceptibility to fake news about it). Soc. Psychol. Personal. Sci. **11**, 1119-1128. (10.1177/1948550620940539)

[28] Scheyder E, Brown N. 2020 ‘Anyone can get it,’ Trump supporters shocked at diagnosis, unwavering in support.

[29] Georgiou N, Delfabbro P, Balzan R. 2020 COVID-19-related conspiracy beliefs and their relationship with perceived stress and pre-existing conspiracy beliefs. Pers. Individ. Dif. **166,** 110201. (10.1016/j.paid.2020.110201) 32565592 PMC7296298

[30] van Prooijen JW. 2017 Why education predicts decreased belief in conspiracy theories. Appl. Cogn. Psychol. **31**, 50-58. (10.1002/acp.3301) 28163371 PMC5248629

[31] Schneider CR, Dryhurst S, Kerr J, Freeman ALJ, Recchia G, Spiegelhalter D, van der Linden S. 2021 COVID-19 risk perception: a longitudinal analysis of its predictors and associations with health protective behaviours in the United Kingdom. J. Risk Res. **24**, 1-20. (10.1080/13669877.2021.1890637)

[32] Galliford N, Furnham A. 2017 Individual difference factors and beliefs in medical and political conspiracy theories. Scand. J. Psychol. **58**, 422-428. (10.1111/sjop.12382) 28782805

[33] Jolley D, Douglas KM, Skipper Y, Thomas E, Cookson D. 2021 Measuring adolescents' beliefs in conspiracy theories: development and validation of the adolescent conspiracy beliefs questionnaire (ACBQ). Br. J. Dev. Psychol. **39**, 499-520. (10.1111/bjdp.12368) 33556990

[34] Faul F, Erdfelder E, Buchner A, Lang A-G. 2009 Statistical power analyses using G*Power 3.1: tests for correlation and regression analyses. Behav. Res. Methods **41**, 1149-1160. (10.3758/BRM.41.4.1149) 19897823

[35] Mason J, Holland S. 2020 Trump says catching COVID-19 was ‘blessing from god’. *Reuters*, 7 October 2020. See https://www.reuters.com/article/health-coronavirus-trump-int-idUSKBN26S2KM.

[36] Stanley M, Barr N, Peters K, Seli P. 2020 Analytic-Thinking Predicts Hoax Beliefs and Helping Behaviors in Response to the COVID-19 Pandemic. *PsyArXiv* March 30. (10.31234/osf.io/m3vth)

[37] Stanley ML, Barr N, Peters K, Seli P. 2021 Analytic-thinking predicts hoax beliefs and helping behaviors in response to the COVID-19 pandemic. Think. Reason. **27**, 464-477. (10.1080/13546783.2020.1813806)

[38] Maertens R, Götz F, Schneider CR, Roozenbeek J, Kerr JR, Stieger III S, Drabot K, van der Linden S. 2021 The Misinformation Susceptibility Test (MIST): A psychometrically validated measure of news veracity discernment.

[39] Bauer DJ, Curran PJ. 2005 Probing interactions in fixed and multilevel regression: inferential and graphical techniques. Multivariate Behav. Res. **40**, 373-400. (10.1207/s15327906mbr4003_5) 26794689

[40] Miller JM, Saunders KL, Farhart CE. 2016 Conspiracy endorsement as motivated reasoning: the moderating roles of political knowledge and trust. Am. J. Pol. Sci. **60**, 824-844. (10.1111/ajps.12234)

[41] BBC. 2020 Trump Covid: President downplays virus on leaving hospital. See https://www.bbc.co.uk/news/election-us-2020-54427390.

[42] Ugarte DA, Cumberland WG, Flores L, Young SD. 2021 Public attitudes about COVID-19 in response to President Trump's social media posts. JAMA Netw. Open **4**, e210101. (10.1001/jamanetworkopen.2021.0101) 33523187 PMC7851723

[43] Fischer S. 2020 Trump's tweet on positive coronavirus test is his most shared ever. *Axios*, 2 October. See https://www.axios.com/2020/10/02/trump-tweet-coronavirus-positive-most-shared.

[44] Downs A. 1957 An economic theory of democracy. New York, NY: Harper.

[45] CNN. 2020 A timeline of Trump's battle with Covid-19. 12 October. See https://edition.cnn.com/interactive/2020/10/politics/trump-covid-battle/.

[46] Finucane ML, Slovic P, Mertz CK, Flynn J, Satterfield TA. 2000 Gender, race, and perceived risk: the ‘white male’ effect. Heal. Risk Soc. **2**, 159-172. (10.1080/713670162)

[47] van der Linden S, Roozenbeek J, Compton J. 2020 Inoculating against fake news about COVID-19. Front. Psychol. **11**, 566790. (10.3389/fpsyg.2020.566790) 33192844 PMC7644779

[48] van Stekelenburg A, Schaap G, Veling H, Buijzen M. 2021 Investigating and improving the accuracy of US Citizens' beliefs about the COVID-19 pandemic: longitudinal survey study. J. Med. Internet Res. **23**, e24069. (10.2196/24069) 33351776 PMC7806340

[49] Imhoff R, Lamberty P. 2020 A bioweapon or a hoax? The link between distinct conspiracy beliefs about the coronavirus disease (COVID-19) outbreak and pandemic behavior. Soc. Psychol. Personal. Sci. **11**, 1110-1118. (10.1177/1948550620934692) PMC7342934 38602949

[50] Tanase L-M, Kerr J, Freeman ALJ, Schneider CR. 2022 COVID-19 risk perception and hoax beliefs in the US immediately before and after the announcement of President Trump's diagnosis. *Figshare*. (10.6084/m9.figshare.c.6123514) PMC9346356 35950194

